# Identifying core strategies and mechanisms for spreading a national medicines optimisation programme across England—a mixed-method study applying qualitative thematic analysis and Qualitative Comparative Analysis

**DOI:** 10.1186/s43058-022-00364-5

**Published:** 2022-10-29

**Authors:** Alexandra Ziemann, Andrew Sibley, Sam Tuvey, Sarah Robens, Harry Scarbrough

**Affiliations:** 1 Centre for Healthcare Innovation Research, City, University of London, Northampton Square, London, EC1V 0HB UK; 2 Department for Social & Policy Sciences, University of Bath, Building 3 East, Claverton Down, Bath, BA2 7AY UK; 3 Wessex Academic Health Science Network, 2 Venture Road, Southampton, SO16 7NP UK; 4 South West Academic Health Science Network, Vantage Point, Pynes Hill, Exeter, EX2 5FD UK; 5 Re!nstitute, Six Landmark Square, Suite 400, Stamford, CT 06901 USA; 6 Bayes Business School, City, University of London, Northampton Square, London, EC1V 0HB UK

**Keywords:** Spread, Innovation, Strategies, Mechanism, Function, Core components, Medicines, Qualitative Comparative Analysis

## Abstract

**Background:**

Achieving widespread adoption of innovations across health systems remains a challenge. Past efforts have focused on identifying and classifying strategies to actively support innovation spread (replicating an innovation across sites), but we lack an understanding about the mechanisms which such strategies draw on to deliver successful spread outcomes. There is also no established methodology to identify core strategies or mechanisms which could be replicated with fidelity in new contexts when spreading innovations. We aimed to understand which strategies and mechanisms are connected with successful spread using the case of a national medicines optimisation programme in England.

**Methods:**

The study applied a comparative mixed-method case study approach. We compared spread activity in 15 Academic Health Science Networks (AHSN) in England, applied to one innovation case, Transfers of Care Around Medicines (TCAM). We followed two methodological steps: (1) qualitative thematic analysis of primary data collected from 18 interviews with AHSN staff members to identify the strategies and mechanisms and related contextual determinants and (2) Qualitative Comparative Analysis (QCA) combining secondary quantitative data on spread outcome and qualitative themes from step 1 to identify the core strategies and mechanisms.

**Results:**

We identified six common spread strategy-mechanism constructs that AHSNs applied to spread the TCAM national spread programme: (1) the unique intermediary position of the AHSN as “honest broker” and local networking organisation, (2) the right capacity and position of the spread facilitator, (3) an intersectoral and integrated stakeholder engagement approach, (4) the dynamic marriage of the innovation with local health and care system needs and characteristics, (5) the generation of local evidence, and (6) the timing of TCAM. The QCA resulted in the core strategy/mechanism of a timely start into the national spread programme in combination with the employment of a local, senior pharmacist as an AHSN spread facilitator.

**Conclusions:**

By qualitatively comparing experiences of spreading one innovation across different contexts, we identified common strategies, causal mechanisms, and contextual determinants. The QCA identified one core combination of two strategies/mechanisms. The identification of core strategies/mechanisms and common pre-conditional and mediating contextual determinants of a specific innovation offers spread facilitators and implementers a priority list for tailoring spread activities.

**Supplementary Information:**

The online version contains supplementary material available at 10.1186/s43058-022-00364-5.

Contributions to the literature

- We know what strategies support the spread of innovations across health systems, but we do not know much about the mechanisms which such strategies draw on to deliver successful spread outcomes.
- Spread success has been linked to replicating core components of an innovation or strategy, but there is no established approach to determine the core components.
- By comparing the experiences of spreading one innovation across different contexts, we identified common strategies/mechanisms and pre-conditional and mediating contextual determinants to inform the tailoring of spread activity.
- The Qualitative Comparative Analysis identified one core combination of two strategies/mechanisms to be prioritised when replicating TCAM/similar innovations.

## Background

Achieving widespread adoption of innovations in health and social care systems remains a challenge [1]. While some innovations seem to find their way across a system almost organically, many are stuck in a state of pilot implementation, are only ever used by one or a few sites, and fail to replicate in other sites [1–3]. This replication problem is grounded in a mismatch of a complex innovation and a complex implementation context which can differ widely between implementation sites and is also subject to dynamic changes over time [1, 4, 5]. This highlights the need for strategies to actively facilitate the widespread adoption of innovations in different sites [1, 5, 6].

The concept of spreading innovations has been described as under-theorised and lacking a commonly accepted definition [7–9]. The term spread is often used interchangeably with scale-up, diffusion, or dissemination [7, 8, 10, 11]. We are defining spread in this study as an active and planned process to replicate and achieve the adoption of an innovation in several sites or organisations for the benefit of a larger population [12]. We understand spread to encompass both the active system-level diffusion *across* adopting organisations/sites and localised, organisational implementation processes *within* such organisations/sites [3, 8, 13].

Strategies have been defined in the implementation science field as “methods or techniques used to improve adoption, implementation, sustainment, and scale-up of interventions” [14 , p.2]. In this study, we are focusing on strategies used to achieve spread. There has been a lot of work to describe and categorise strategies in implementation science. For example, the seminal Expert Recommendations for Implementing Change (ERIC) project has identified 73 different strategies in nine clusters encompassing engaging consumers, developing stakeholder relationships, using evaluative and iterative strategies, financial strategies, changing infrastructure, adaptation and tailoring to the context, providing interactive assistance, and training and educating stakeholders [15–17]. Such taxonomies have helped consolidate our understanding of what strategies are used. However, most studies on spread efforts are descriptive, and there is a lack of evidence explaining how a strategy leads to successful spread [8, 14], and a paucity of comparative studies seeking to identify strategies which might be more effective in leading to successful spread [8, 18, 19].

Against this backdrop, one way forward in determining how certain strategies lead to successful outcomes involves focussing on the underlying functions or causal mechanisms of strategies, alongside the interaction of those strategies with the specific context in which they are applied [8, 14, 20]. Mechanisms have been defined by Lewis et al. as a “process or event through which an implementation strategy operates to affect desired implementation outcomes” ([21] , p.2). While we have a good theoretical understanding of which mechanisms are underlying strategies, in practice which mechanisms are activated and lead to an implementation outcome depends on the context, including the (type of) innovation [20, 22]. Lewis and colleagues highlight that there is a need for conceptual clarity and methodological advancements in mechanism-focused implementation research. As such, they encourage the use of qualitative methods to advance our understanding of causal mechanisms beyond the more descriptive approach adopted by many studies in this field [21].

Identifying and understanding the underlying mechanisms of strategies could also lead to more generalisable knowledge to support the spread of innovations. There has been a lot of discussion around the importance of identifying what is the core of an innovation or strategy [23–25]. This core would be defined as being necessary for an innovation or strategy to work, and thus, the core would be the generalisable aspects which are to be replicated in new contexts while peripheral aspects could be adapted to fit the local context [20, 23, 24, 26]. The observable activities associated with a strategy or its form (e.g. a training workshop for stakeholders) could be such an adaptable or peripheral aspect, but the underlying mechanisms (e.g. building capacity of stakeholders) could be the generalisable or core component. There is no common definition of what core components of spread efforts are, nor is there an established approach to identify them [25, 26]. Comparative methodologies could provide a useful methodological approach to identify the core mechanisms [27]. In particular, configurational comparative methods might offer the opportunity to identify core components, and more importantly a combination of components, by prioritising necessary and sufficient components that are linked to a successful outcome [28, 29].

We aimed to add empirical evidence to understand which spread strategies and mechanisms lead to successful spread using the case of a national medicine optimisation programme which was spread across 15 regions in England. We also aimed at exploring how a combination of qualitative and configurational comparative mixed methodology can support the identification of mechanisms, their interplay with contextual determinants, and core spread strategies and mechanisms. We focused our analysis on spread strategies applied by Academic Health Science Networks (AHSN) which are local/regional intermediary organisations with the official mandate to facilitate the spread of innovations across the National Health System (NHS) in England [30]. As AHSNs have been largely free to develop their own spread strategies, they provide a unique “natural laboratory” to compare the different strategies applied to spreading the same innovation in multiple contexts.

## Methods

### Design

The study applied a comparative mixed-method case study approach drawing on primary qualitative and secondary quantitative data. We followed two methodological steps: (1) qualitative thematic analysis of primary data collected from semi-structured interviews and (2) crisp-set Qualitative Comparative Analysis (QCA) [29, 31], using secondary quantitative data on spread outcome and the qualitative themes resulting from step 1. We are reporting on step 1 according to the Consolidated Criteria for Reporting Qualitative Research (COREQ) guidelines, and the COREQ checklist can be found in Additional file 1. There are no official standards for reporting research applying QCA (step 2); however, we have applied the standards of good practice in QCA as suggested by Schneider and Wagemann [32]. The study is part of a wider study “Review of Spread and Adoption Approaches across the AHSN Network” which explored the general spread activity at AHSNs based on 143 interviews with AHSN staff members [33]. Ethical approval for this study was obtained from City, University of London, The Business School Research Ethics Committee (ETH1920-1032).

### Setting

The study focused on analysing spread strategies applied by the staff working at AHSNs in England. AHSNs were set up by NHS England in 2013 and relicensed in April 2018 to operate as the key innovation arm of the NHS [30]. They are intermediary organisations designed to connect NHS organisations with academic organisations, local authorities, the third sector, and industry. Their role is to support their local health and social care system to adopt innovations at pace and scale, improve population health, and generate economic growth. There are 15 AHSNs in England that are each covering a distinct geography and are organised in a national umbrella network, the AHSN Network. All AHSNs have the same commissioners (NHS England and NHS Improvement and the United Kingdom Government’s Office for Life Sciences) and follow a broadly similar pattern of innovation activity. This was described by Ferlie et al. as “scouting innovations, promoting evidence-based innovations, building relationships and matchmaking, and cross-institutional regional brokerage and support for regional innovation systems” [34]. AHSNs have been largely free to develop their own spread strategies.

### Case study

We have compared the spread strategies applied by all 15 AHSNs to one innovation case, the Transfers of Care Around Medicines (TCAM) national spread programme. TCAM is one of the seven national spread programmes adopted by the AHSN Network and supported by NHS England to be spread across England between 2018 and 2020, facilitated by the 15 local AHSNs [35, 36]. TCAM’s purpose is to improve the sharing of information about patients’ medicines after hospital discharge with the patients’ dedicated community pharmacy. Community pharmacists can provide a follow-up consultation with the patient to support them in taking their (potentially changed) medication with the aim to reduce the risk of re-admission to hospital or emergency department attendance. TCAM is supporting acute hospital trusts to implement a software solution to electronically transfer information with the patient’s consent to community pharmacists. It was originally developed and piloted in the Northeast of England and further developed by the AHSNs in Wessex and the West of England before it became a national spread programme.

### Step 1: qualitative thematic analysis

Between March and June 2020, we conducted semi-structured interviews with the AHSN staff on the spread process of TCAM from all 15 AHSNs in England.

#### Participant selection and recruitment

Interview participants were purposively selected based on their expertise and experience in being involved in operational or senior management roles in the spread of TCAM. At each AHSN, the research team had (senior) management contacts who nominated participants for interviews and provided contact details of participants to the research team. Recruitment of participants was conducted by AS, AZ, or ST via email or telephone.

#### Data collection

Individual one-to-one interviews were conducted by AZ and ST via phone in a remote working/home office context (interviews were conducted during the COVID-19 pandemic under a work-from-home-if-possible advice by the United Kingdom Government that applied to research team members and participants). The interviews were conducted applying a semi-structured interview guide asking for participants’ activity and experiences spreading TCAM during the same 2-year period between 1 January 2018 and 31 December 2019. The open-ended interview guide was developed covering key causal implementation pathway aspects; context, strategies and mechanisms, and outcomes [19]. Interview questions were enquiring about the development and application of spread activities including the application of scientific theory, contextual determinants of spread with a specific focus on national-level determinants, and lessons learned including examples for successful and unsuccessful spread cases (Additional file 2). The interview guide was pilot tested by AS with the staff at Wessex AHSN who were not participants. Interviews were audio-recorded and transcribed verbatim.

#### Data analysis

Data from interviews were analysed by applying qualitative thematic analysis. Interviews were coded inductively by AS, AZ, and ST using NVivo (version March 2020) [37]. We extracted first-order themes and relevant quotes for spread strategies and contextual determinants. Data extraction for spread strategies was structured by broad categories derived from two implementation strategy frameworks by Powell et al. [16] and Leeman et al. [13]. Data on contextual determinants was structured by three categories derived from the Consolidated Framework for Implementation Research [38] describing the characteristics of the innovation (TCAM), the outer context (differentiated into local/regional and national contexts), and individual stakeholder characteristics. The coding tree can be found in Additional file 3. Data extraction was conducted by AZ, AS, and ST. To ensure the trustworthiness and validity of our analysis, we used a de-briefing technique with one research team member conducting the initial analysis for one AHSN which was discussed in the group until a consensus was reached.

#### Data synthesis

First-order themes around spread strategies and contextual determinants and quotes from interviews were synthesised by AZ in the form of case descriptions for each AHSN. From the cross-case analysis, AZ derived second-order themes on common spread strategies and mechanisms and contextual determinants. These were synthesised in narrative form, and their interlinkages were illustrated in the form of a simplified causal diagram [39, 40]. Contextual determinants were categorised based on the qualitative data into pre-conditions, mediators, or moderators [20]. The synthesis of findings including quotes was returned to the participants for feedback and a few comments around the potential to identify participants or sites from case descriptions and quotes were addressed.

### Step 2: Qualitative Comparative Analysis

In this study, we applied QCA to provide an additional systematic layer of analysing the findings from the qualitative thematic analysis (step 1). We aimed at identifying core AHSN spread strategies and mechanisms (or combinations thereof) that are connected to the successful spread of TCAM [41].

We chose crisp-set QCA because of the small number, case-based, and mixed data which was defined in a dichotomised way as the “absence” or “presence” of spread strategies and mechanisms (conditions) in the set of all 15 AHSNs (cases) which represented either the successful or unsuccessful spread of TCAM (outcome). The QCA was based on qualitative themes for spread strategies and mechanisms derived from the qualitative thematic analysis in step 1 and quantitative TCAM spread outcome data officially reported by each AHSN to the AHSN Network National Metrics Dashboard [42] (Additional file 4).

The initial input to the QCA was defined with a value of 1 (theme/condition present in this AHSN) and 0 (theme/condition absent in this AHSN). We defined the presence of a condition when at least one interviewee mentioned once that their AHSN applied that strategy/mechanism. The outcome for each AHSN is based on their formal reports of the adoption rate at the end of the calendar year 2019 (QCA code: OUTCOME) with a value of 1 (successful case = adoption rate 50% or higher) and a value of 0 (unsuccessful case = 49% or lower), as defined by the AHSN Network. The values of the conditions are formulated as being connected with a successful outcome (as opposed to an unsuccessful outcome).

The quality of the initial truth table based on the raw data table which included all themes and sub-themes as conditions was assessed based on the established quality criteria [31]. These were a variety of values across cases, conditions and outcomes, avoidance of any contradictory configurations of conditions, and confidence in the quality of the qualitative data to reliably represent the absence/presence of a theme in each case. To meet the quality criteria, we excluded conditions which led to a refinement of the truth table with the final set of cases, configurations of conditions, and outcomes as input for the Boolean minimization.

For the minimisation processes, we conducted four analyses, two for configurations with a positive and a negative outcome, and two either including or excluding logical remainders (i.e. configurations without observed cases). The minimisation resulted in complex and parsimonious solution pathways of necessary and/or sufficient strategies and mechanisms for a positive spread outcome. As all configurations of conditions were tenable, we did not report an intermediate solution based on a selection of logical remainder configurations. Sufficiency has been defined as if always when the condition/solution is present, the outcome is also present. Necessity has been defined as if always when the outcome is present, the condition/solution is also present [32]. We used TOSMANA version 1.6.1.0 for the QCA [43].

We provide a statement on the research team and reflexivity in Additional file 5.

## Results

### Study participants

We interviewed 18 participants from all 15 AHSNs of which four were senior management staff and 14 were the operational staff. No participant dropped out or refused to participate. Interviews lasted 60 min on average.

### Qualitative thematic analysis

**Fig. 1:**
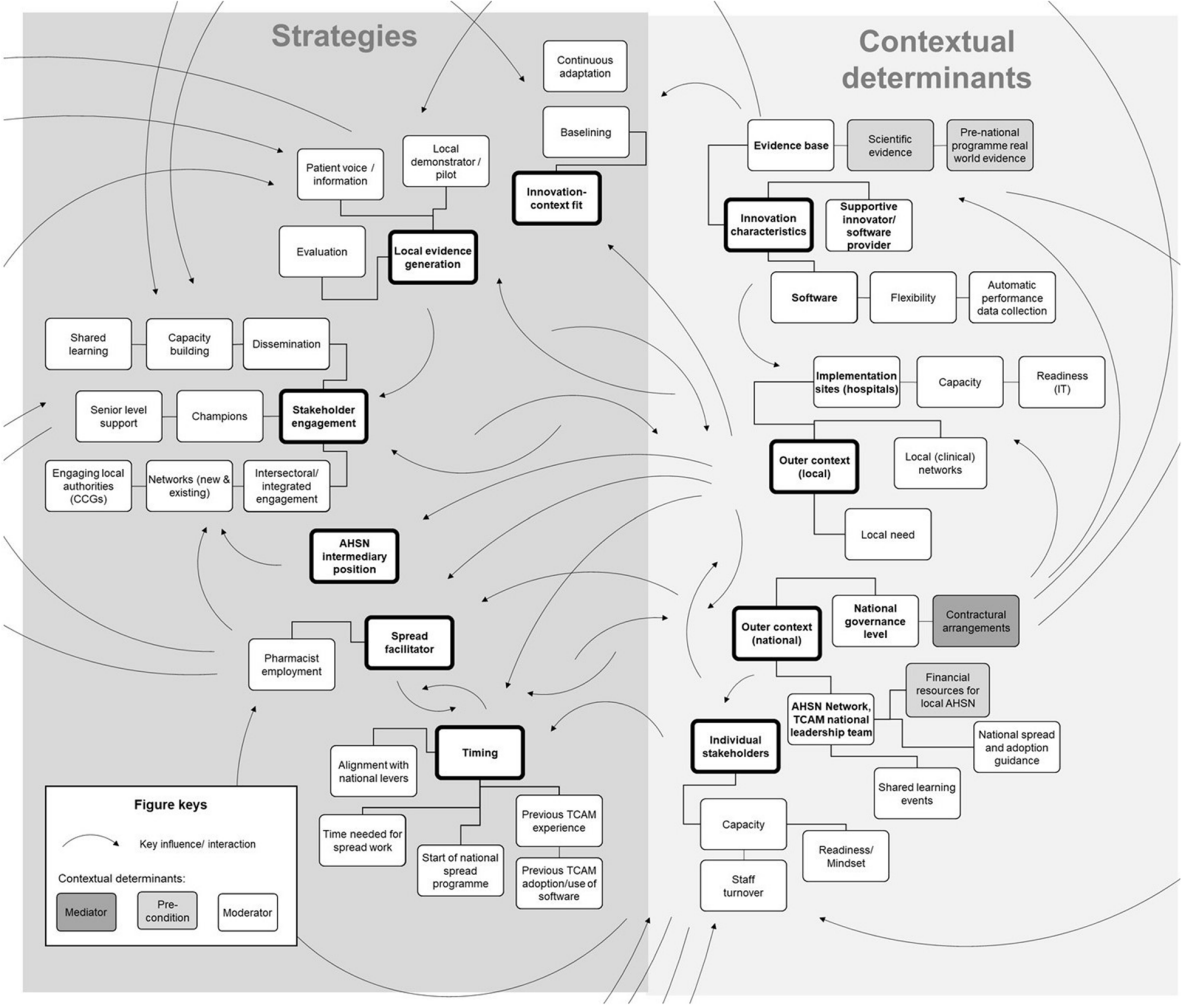
Causal diagram showing the connection between TCAM spread strategies/mechanisms and contextual determinants. The causal diagram shows the connection between cross-case themes for contextual determinants on the right (light grey) and themes for spread strategies and mechanisms on the left (dark grey). Themes for strategies and mechanisms are grouped by second-order theme and divided into sub-categories of key first-order themes for spread strategies. Themes for contextual determinants are grouped following the Consolidated Framework for Implementation Research (CFIR) [33] into outer contextual factors differentiated by national and local levels, characteristics of the innovation (TCAM), and individual stakeholder characteristics. Sub-categories of contextual determinants were classified as either pre-condition, moderator, or mediator explaining further how they are influencing strategies/mechanisms [19]. Arrows are showing the connection or influence between the groups (not between specific sub-categories). A clear mediator was the inclusion of TCAM into standard contracts at the end of the national programme, making it de facto mandatory to implement TCAM for adopters which reportedly started to increase the adoption rate and impacted the effectiveness or need for spread strategies. There are a few contextual factors that were classified as pre-conditions such as financial resources provided to AHSNs to fund spread strategies and scientific and real-world evidence about TCAM which were the backbone of dissemination and capacity-building activities. The other contextual factors can be seen as moderators affecting the speed and success of spread strategies such as, for example, availability of implementation guidance provided by the national leadership team, the availability of a local need for TCAM, the level of capacity and readiness of adopters for TCAM (both of organisations and individual stakeholders), the flexibility of the TCAM software allowing for adaptation, and the possibilities to share learning among spread facilitators

The case descriptions for each AHSN are presented in summary form in Additional file 6. In the following, we present key themes on strategies and mechanisms from the cross-case analysis and show how they were connected with themes for contextual determinants, classified as pre-conditions, mediators, and moderators, in the causal diagram (Fig. 1). To clarify the link to the QCA, we are including the codes for QCA conditions in brackets after each sub-theme.

#### Unique intermediary position of the AHSN as “honest broker” and local networking organisation

AHSNs facilitate spread by supporting commercial and non-commercial innovators and health and care system adopters from invention through to adoption and post-adoption evaluation in practice. They take a unique intermediary position that was mentioned by many interviewees as advantageous to achieve a successful spread. They gained the trust of local/regional health system stakeholders as they were not following a competing agenda of their own and were able to function as moderators to gather all stakeholders together around a common goal of spreading an innovation.

We are at arm’s length, we’re an equal partner, we’re an honest broker [ … ], we’re not a commissioner, we’re not a performance manager, that enables them to create relationships of trust and credibility and support. We move around all of the system. Operational staff member, AHSN 7

#### Right expertise, experience, and position of the spread facilitator

A key theme emerged around the expertise, experience, and position of the AHSN staff member leading the spread of TCAM. Employing a locally embedded pharmacist for the spread of this medicine optimisation programme on a part-time basis at the AHSN offered several advantages (QCA code: PHARMA). These clinical leads could gain a head start by understanding the innovation and potential barriers to adoption, knowing the local context, speaking the same language with adopting pharmacists in trusts and in the community, already having built the relationships to the different stakeholders working in the local health and care system, and if in a senior position, they already gained the respect of the stakeholders.

They’d became aware that they couldn’t just run this with the project manager. They needed to have the pharmacy teams. That’s why I was called in […] to then build those relationships. Operational staff member, AHSN 15

Employing a spread facilitator in a part-time role at the AHSN (and having the financial resources to do so) was separated from the role of local voluntary champions (QCA code: CHAMP). The employment gave them the thinking space, time, and legitimacy to lead on spreading the innovation which they might not have in their day job.

#### Intersectoral and integrated stakeholder engagement

Stakeholder engagement can be seen as a key spread strategy for AHSNs. We identified themes around particularly effective mechanisms of engagement evolving around intersectoral (QCA code: INTERSECT) and integrated engagement strategies such as building on existing or establishing new local networks (QCA code: NETWORK) of ideally all stakeholders involved in a particular spread programme and early in the spread programme. Other important themes that emerged in this regard were securing senior support in the local health and care system and engaging local authorities to support the spread effort (QCA code: SENIOR) and involving local health system decision-making and financing bodies, the Clinical Commissioning Groups (CCG, QCA code: CCG).

The key stages with my implementation have been getting the right people involved at the start of the project. […] I’ve established a TCAM community of practice […] and it’s got a representative from [the different stakeholder organisations]. It’s actually allowed us to [have] confident and strong conversations. Operational staff member, AHSN 6

AHSNs were provided with the financial resources to organise events for and regular meetings of the networks and use these for dissemination activities, sharing learning, and capacity building. A key moderator of stakeholder engagement was the capacity and readiness of individual stakeholders.

#### Dynamically marrying the innovation with local health and care system needs and characteristics

AHSNs had to be flexible and adapt to meet the (changing) needs and characteristics of the local context specific to a particular innovation (QCA code: CONTEXT). A common spread strategy evolved called “baselining”, a comprehensive assessment exercise at the start of the spread programme to understand the innovation and its interlinkages with the local context including a stakeholder analysis. This innovation-context assessment would have usually led to adaptations of the innovation itself or the way in which it is delivered or implemented or, in some cases, to the decision that the local system is either not in need or not ready in which case the TCAM implementation would be halted. The flexibility offered by the innovation in terms of the different software integration models and by the innovator and software provider in terms of their openness to change and further development of the software enabled local adaptation.

So you can’t have a blanket approach. […] As much as you might have a one-size-fits all project, there are going to be local variances and local barriers that we need to address, and part of our role is […] to try to come to […] a win-win for all. Operational staff member, AHSN 14

A key mechanism was the flexibility of the spread strategies themselves which AHSNs would dynamically adapt in the course of the spread process if the context-innovation fit changed.

#### Generating local evidence

Next to scientific evidence around the effectiveness of the innovation and evidence around how the innovation works in the real world, local effectiveness and impact of the innovation and implementation strategies were of particular importance in engaging local adopters. Evidence was often challenged if it was generated in another region resulting in the request for a pilot study or “local demonstrator” to produce this local evidence as part of evaluating the local implementation which would be financially supported by the local AHSNs (QCA code: PILOT). The automatic provision of performance readings in the TCAM software was often mentioned as enabling local evaluation. Furthermore, incorporating the patient’s voice was rated as a valuable additional source of evidence. While the generation of localised evidence seemed key for spread, the AHSN staff realised that there was an emerging risk of running too many local pilots, which would waste time and resources. This effect was often referred to as “pilotitis”, and some interviewees reported starting to push against adopter requests for pilots especially where local real-world evidence exists from elsewhere.

There are always the people who want to see evidence that’s been generated in their area. […] Pilotitis [sic] which is, [in this area] we’ll pilot it again. […] Then, we’ll make a decision. Rather than […] trusting evidence which has been generated elsewhere and assuming that will be transferrable […]. Operational staff member, AHSN 2

#### Timing of TCAM

Some themes emerged around the timing of TCAM. TCAM was developed, pilot-tested, and implemented in a few AHSN areas before it was spread nationally (QCA code: ADOPT). Some AHSNs reported an advantage by already having started local implementation at the beginning of the national programme. Other AHSNs reported a disadvantage due to a history of negative experiences with previous TCAM implementation in their area (QCA code: EXP). Another important theme around timing emerged in terms of the starting time of the spread work which was delayed in some AHSNs because of competing commitments and more time needed for “baselining” activities (QCA code: TIMELY). Furthermore, interviewees often mentioned that if they could have waited to spread TCAM until national levers in the form of contractual arrangements mandating the use of TCAM came into force, it would have saved them a lot of time and resources. The timing was often affected by the readiness of local implementation sites, mainly in terms of IT systems.

### Qualitative Comparative Analysis

We compared the following key themes and sub-themes for strategies and mechanisms to identify what is the most important strategy/mechanism of spread success. As part of the timing theme, we compared the components of a timely start into the spread work, previous experiences in terms of having adopted TCAM and having had negative experiences with TCAM. As part of the stakeholder engagement theme, we compared the components of engaging CCGs, engaging senior-level stakeholders, following an intersectoral approach, and engaging existing networks. We also intended to compare the strategies/mechanisms of local champions with employing a pharmacist as a spread facilitator. Two second-order themes, the adaptation to context and local evidence generation in pilot studies, were included as stand-alone themes as we have not identified clear sub-themes. The second-order theme of the unique positioning of AHSNs was not included in the QCA as it was the same for every case. Table 1 shows the raw data table as initial input to the QCA.

**Table 1:** QCA raw data table

			AHSN ID														
			1	2	3	4	5	6	7	8	9	10	11	12	13	14	15
Second-order themes and key sub-themes (QCA condition ID code)	Timing	A timely start of TCAM spread work (TIMELY)	0	0	0	1	1	1	1	1	1	1	1	0	1	1	1
		Prior adoption of TCAM (ADOPT)	1	0	1	0	0	1	0	1	1	1	0	0	1	1	0
		Negative spread experiences (EXP)	0	0	1	0	0	1	0	1	1	0	0	0	1	1	0
	Local evidence	Local pilot studies (PILOT)	0	1	1	1	0	1	0	1	1	1	1	0	1	1	1
	Spread facilitator	Employing a (local, senior) pharmacist at the AHSN (PHARMA)	0	1	1	1	0	1	0	1	1	0	0	1	1	1	1
		Engagement of local champions (CHAMP)	1	0	0	1	0	1	1	1	1	0	0	1	1	1	1
		Securing senior-level support/buy-in (SENIOR)	0	1	1	1	0	1	1	1	1	0	0	1	1	1	1
	Stakeholder engagement	Involvement of CCGs (CCG)	1	1	1	1	0	1	1	1	0	0	0	0	1	1	0
		Intersectoral stakeholder engagement (INTERSECT)	1	1	1	0	1	1	1	0	0	0	1	0	1	1	1
		Spreading through local networks (NETWORK)	1	1	1	1	0	1	1	1	1	0	1	0	1	1	1
	Innovation-context fit	Establishing local innovation-context fit (CONTEXT)	0	0	1	1	1	1	1	1	1	1	1	0	1	1	1
Outcome			0	0	0	1	0	1	0	1	1	0	0	0	1	1	1

After assessing the quality of the initial truth table based on the raw data table, we excluded the following conditions from the QCA based on the lack of variation across the cases: CONTEXT, NETWORK, and EXP. Furthermore, we decided to exclude the following conditions as we were not confident enough that the qualitative data would clearly indicate the presence or absence of these strategies/mechanisms for all cases: CHAMP, CCG, PILOT, and SENIOR. Table 2 shows the final truth table.

**Table 2:** QCA truth table

Number of cases	AHSN ID	TIMELY	ADOPT	PHARMA	INTERSECT	OUTCOME	Consistency
1	12	0	0	1	0	0	0
1	2	0	0	1	1	0	0
1	1	0	1	0	1	0	0
1	3	0	1	1	1	0	0
3	5, 7, 11	1	0	0	1	0	0
1	4	1	0	1	0	1	1
1	15	1	0	1	1	1	1
1	10	1	1	0	0	0	0
2	8, 9	1	1	1	0	1	1
3	6, 13, 14	1	1	1	1	1	1
0		0	0	0	0	L	
0		0	1	0	0	L	
0		0	1	1	0	L	
0		0	0	0	1	L	
0		1	0	0	0	L	
0		1	1	0	1	L	

*L* logical remainder (configurations of conditions without observed cases)

The Boolean minimisation process reduced the possible configurations of conditions and outcomes to complex and parsimonious solutions, separately explaining successful and unsuccessful TCAM spread (Table 3, Additional file 7).

**Table 3:** QCA parsimonious solutions

Outcome	Successful TCAM spread	Unsuccessful TCAM spread	
QCA solution	TIMELY * PHARMA	timely + pharma	
AHSNs covered by solution (ID)	4; 6, 13, 14; 8, 9; 15	1; 2; 3; 12;	1; 5, 7, 11; 10
Raw coverage	1.0	0.5	0.625
Unique coverage	1.0	0.375	0.5
Solution coverage	1.0	1.0	
Solution consistency	1.0	1.0	

Upper case letter = condition is present; lower case letters = condition is absent; * = combination of conditions (Boolean AND); + = non-combined/alternative conditions (Boolean OR)

The solution for success explained 100% of successful cases (raw coverage = 1.0, solution coverage = 1.0) and was the only solution explaining all cases of successful outcome (unique coverage = 1.0, solution consistency = 1). The solution term for unsuccessful TCAM spread contains two alternative pathways. Four unsuccessful AHSNs had a delayed start (pathway 1), and five unsuccessful AHSNs did not have a pharmacist employed to facilitate spread work (pathway 2). One of these AHSNs had both a delay and did not have a pharmacist employed. The solution for unsuccessful outcomes explained all eight unsuccessful cases (solution coverage = 1.0). Each alternative pathway in the solution is covering about half of the cases (raw coverage = 0.5 and 0.625). Each pathway is not solely explaining a number of unsuccessful cases, but one case is covered by both solutions (unique coverage = 0.375 and 0.5). The low level of coverage of the separate pathways or conditions included in the solution explaining unsuccessful TCAM spread confirms the validity of the solution for successful TCAM spread. Only the combination of the two strategies/mechanisms led to successful TCAM spread, and whenever this combination of strategies is absent, the spread of TCAM was unsuccessful (solution consistency = 1, solution coverage = 1.0)

Successful TCAM spread was explained by a timely start of the TCAM spread work combined with a (local, senior) pharmacist facilitating the spread work at the AHSN. Unsuccessful TCAM spread was explained by either the delay of spread work or the absence of a pharmacist facilitating the spread work. The other spread strategies/mechanisms (adoption before the national programme started and an intersectoral engagement approach) were not relevant to explaining TCAM spread outcomes. All seven successful AHSNs had a timely start and employed a pharmacist.

This combination of the two spread strategies/mechanisms can be considered a sufficient combination of conditions for successful TCAM spread. There is no other alternative path leading to success, this combination needs to be present for successful spread to occur, and if it is not present, TCAM spread is unsuccessful. While each separate strategy/mechanism in the solution pathway can be considered necessary for the spread outcome to occur, neither of the two conditions are sufficient for successful TCAM spread on their own.

## Discussion

We identified six common spread strategy-mechanism constructs that were applied by AHSNs to spread the TCAM programme: (1) the unique intermediary position of the AHSN as “honest broker” and local networking organisation, (2) the right capacity and position of the individual “spread facilitator”, (3) an intersectoral and integrated stakeholder engagement approach, (4) the dynamic marriage of the innovation with local health and care system needs and characteristics, (5) the generation of local evidence, and (6) the timing of TCAM. The QCA resulted in the core strategy/mechanism of a timely start into the national spread programme in combination with the employment of a local, senior pharmacist at the AHSN who facilitates the spread work.

It is no surprise that strategies such as stakeholder engagement or adaptation/tailoring to the local context feature in our results as they have been identified as relevant strategies in previous studies of spread or scale-up and taxonomies of strategies [5, 17]. The analysis of mechanisms showed that it was important to engage all stakeholders in an integrated fashion and early on in spread activities. This kind of engagement approach might better meet the needs of spreading innovations across a system comprising several different implementation sites and organisations rather than engaging individuals or a particular group of stakeholders [5]. Similarly, dynamic adaptation/tailoring to a changing local context can be expected to feature more prominently when spreading an innovation across several different contexts and across a complex system which is characterised by continuous change [2, 5].

We have identified key spread strategies and mechanisms referring to the spread facilitator and the facilitating organisation. They might be most related to the category of infrastructure changes, particularly the start of a dissemination organisation, and recruiting for leadership or champions as described in Waltz et al.’s taxonomy of strategies [17]. Our analysis of mechanisms showed the importance of AHSNs being an independent or intermediary organisation which at the same time is a known and trusted local partner. While our findings are in line with other studies showing that external and intermediary organisations support implementation, there is a general lack of studies focusing on the role of intermediary organisations in innovation implementation and particularly scale-up and spread of health innovations, as most studies focus on individuals [44, 45]. In terms of the individual spread facilitator, the mechanism was characterised as the right match in terms of clinical expertise and local position and reputation. This particularly underlines the importance of professional background when spreading clinical innovations [18, 46] and confirms Ferlie et al.’s previous results about knowledge leaders at AHSNs who they found to combine professional specialisation and understanding of the local system, rather than relying on more general expertise related to knowledge brokering or implementation [34].

Our study showed how important different dimensions of timing were, with particular reference to the start of local TCAM spread work. Temporality has been described as an important dimension of implementation strategies in terms of their order or sequence [47]. Ilott et al., for example, highlighted further temporal dimensions in their spread case study such as path dependency, alignment with external influences, and implementation work taking time [48], and these are reflected in our findings. Timing does not feature in any taxonomy as a separate strategy. This might be explained by timing being seen as a common-sense activity or something out of control of those spreading innovations. Future studies might want to explore further what role timing plays and if it could indeed be defined as a separate spread strategy/mechanism.

Generating additional local evidence seems to be a strategy not identified in previous taxonomies. Waltz et al. for example refer to using evaluative strategies [17], as do Milat et al. who highlight the importance of the systematic use of evidence throughout spread work [5]. The important mechanism in our study, however, was the generation of new local evidence about the innovation which was seen as essential for overcoming the not-invented-here challenge [1, 49]. The evidence base of an innovation has previously been identified as an important determinant to spread success as characteristic of the innovation [33], as has the relevance of local or contextualised evidence to build confidence among stakeholders for local implementation [50, 51]. Spread strategies have been defined around disseminating this evidence base or using it in capacity-building activities, as occurred in the case of TCAM as well. In the case of this local evidence-generating strategy, the evidence base is adapted or enhanced. Thus, this can be identified as a distinct spread strategy as conceptualised by Rhodes and Lancaster in terms of shifting from evidence-based to evidence-making interventions [52].

The qualitative thematic analysis helped identify common contextual determinants influencing spread strategies and mechanisms in the case of TCAM. Understanding which contextual factors are pre-conditions, moderators, and mediators can inform future spread efforts. In particular, we observed the mediating role of introducing TCAM into standard contracts and thus making it de facto mandatory for adopters to implement it. Towards the end of the national spread programme, this began to have a profound impact on the spread process and reportedly changed the need for, and application of, some of the spread strategies. This shows the importance of further classifying contextual factors and understanding their connection and influence on spread strategies going beyond merely identifying barriers and enablers [19]. It furthers our general understanding about what kind of contextual factors might be more often classified as mediators (e.g. contractual arrangements) and pre-conditions (e.g. funding, evidence), and how they can be differentiated from the usually identified moderators or barriers and enablers. Pre-conditions and mediators might have a more important influence on spread success than moderators and might be prioritised for tailoring strategies or planning spread efforts.

The comparative design of the study has helped us to identify key common and core components of spread strategies/mechanisms. The application of QCA allowed us to identify a combination of strategies/mechanisms that were more important than others in leading to success and that were in place in all successful spread cases and absent in unsuccessful cases. Configurational comparative methods such as QCA are increasingly recognised in implementation science to explain complex causal relationships in real-world settings [28, 53]. As Yakovchenko et al. found in their analysis of implementation strategies using configurational comparative methods, these methods have the potential to offer insights into the causal effects of a combination of strategies instead of analysing single strategies [41].

Additional file 8 discusses further the practice and policy implications.

### Limitations

Our results point towards QCA being a useful methodology for identifying core components. Our QCA results were valid and highly consistent for the four strategies we could compare, but more insight could have been gained if we had been able to include further/all themes for strategies/mechanisms from the qualitative thematic analysis in the QCA. We had to exclude strategy/mechanism themes from the QCA due to the limited variation of values across cases and reliability of qualitative data for each case, some of which might have turned out to be core components. Future studies should explore further the usefulness of QCA or other configurational comparative methods for identifying core components of health innovations and strategies/mechanisms.

Our analysis was based on a relatively small number of interviews which could influence the validity and representativeness of the findings. This study was part of a larger study on general spread strategies at AHSNs (not only related to TCAM) which was based on 143 interviews [33]. This analysis has confirmed the themes around spread strategies presented in this study increasing our confidence in the findings.

## Conclusions

By qualitatively comparing the experiences of spreading one innovation across different contexts, we identified the common strategies, underlying causal mechanisms which strategies draw on, and related contextual determinants differentiated as pre-conditions, mediators, and moderators. The QCA has clearly identified one core strategy/mechanism combination for the TCAM national programme as a timely start into the national programme combined with the employment of a local, senior pharmacist as a spread facilitator. The identification of core strategies/mechanisms and common pre-conditional and mediating contextual determinants offers policymakers and practitioners involved in facilitating spread and/or implementing a specific innovation a priority list for tailoring spread activities.

## Supplementary Information

**Additional file 1.** COREQ checklist.

**Additional file 2.** Interview guide.

**Additional file 3.** Coding tree.

**Additional file 4.** TCAM spread outcomes.

**Additional file 5.** Research team and reflexivity.

**Additional file 6.** Case descriptions.

**Additional file 7.** Detailed QCA results.

**Additional file 8.** Policy and practice implications.

## Data Availability

The datasets used and/or analysed during the current study are available from the corresponding author upon reasonable request.

## Abbreviations

AHSN: Academic Health Science Network
CCG: Clinical Commissioning Groups
CFIR: Consolidated Framework for Implementation Research
COREQ: Consolidated Criteria for Reporting Qualitative Research
ERIC: Expert Recommendations for Implementing Change
ID: Identifier
IT: Information technology
NHS: National Health System
QCA: Qualitative Comparative Analysis
TCAM: Transfer of Care Around Medicines

## References

[1] Horton T, Illingworth J, Warburton W (2018). The spread challenge.

[2] Greenhalgh T, Wherton J, Papoutsi C, Lynch J, Hughes G, A’Court C (2017). Beyond adoption: a new framework for theorizing and evaluating nonadoption, abandonment, and challenges to the scale-up, spread, and sustainability of health and care technologies. J Med Internet Res.

[3] Scarbrough H, Kyratsis Y. From spreading to embedding innovation in health care: implications for theory and practice. Health Care Manag Rev. 2021. 10.1097/HMR.0000000000000323. 10.1097/HMR.0000000000000323 PMC9162066 34319279

[4] Hemmings NHR, Castle-Clarke S, Palmer W (2020). Achieving scale and spread. Learning for innovators and policy-makers.

[5] Milat AJ, Bauman A, Redman S (2015). Narrative review of models and success factors for scaling up public health interventions. Implement Sci.

[6] Chambers DA, Norton WE (2016). The adaptome: advancing the science of intervention adaptation. Am J Prev Med.

[7] Ben Charif A, Zomahoun HTV, LeBlanc A, Langlois L, Wolfenden L, Yoong SL (2017). Effective strategies for scaling up evidence-based practices in primary care: a systematic review. Implement Sci.

[8] Ovretveit J (2011). Widespread focused improvement: lessons from international health for spreading specific improvements to health services in high-income countries. Int J Qual Health Care.

[9] Ilott I, Gerrish K, Pownall S, Eltringham S, Booth AJ (2013). Exploring scale-up, spread, and sustainability: an instrumental case study tracing an innovation to enhance dysphagia care. Implement Sci.

[10] Côté-Boileau É, Denis J-L, Callery B, Sabean M (2019). The unpredictable journeys of spreading, sustaining and scaling healthcare innovations: a scoping review. Health Res Policy Syst.

[11] Shaw J, Tepper J, Martin D (2018). From pilot project to system solution: innovation, spread and scale for health system leaders. BMJ Leader.

[12] NHS England (2022). Glossary. Leading the spread and adoption of innovation and improvement: a practical guide.

[13] Leeman J, Birken SA, Powell BJ, Rohweder C, Shea CM (2017). Beyond “implementation strategies”: classifying the full range of strategies used in implementation science and practice. Implement Sci.

[14] Powell BJ, Fernandez ME, Williams NJ, Aarons GA, Beidas RS, Lewis CC (2019). Enhancing the impact of implementation strategies in healthcare: a research agenda. Front Public Health.

[15] Powell BJ, Waltz TJ, Chinman MJ, Damschroder LJ, Smith JL, Matthieu MM (2015). A refined compilation of implementation strategies: results from the expert recommendations for implementing change (ERIC) project. Implement Sci.

[16] Powell BJ, McMillen JC, Proctor EK, Carpenter CR, Griffey RT, Bunger AC (2012). A compilation of strategies for implementing clinical innovations in health and mental health. Med Care Res Rev.

[17] Waltz TJ, Powell BJ, Matthieu MM, Damschroder LJ, Chinman MJ, Smith JL, et al. Use of concept mapping to characterize relationships among implementation strategies and assess their feasibility and importance: results from the expert recommendations for implementing change (ERIC) study. Imp Sci. 2015;10:109. 10.1186/s13012-015-0295-0 PMC4527340 26249843

[18] De Silva D (2014). Spreading improvement ideas. Tips from empirical research. Evidence scan 20.

[19] Lau R, Stevenson F, Ong BN, Dziedzic K, Treweek S, Eldridge S (2015). Achieving change in primary care—effectiveness of strategies for improving implementation of complex interventions: systematic review of reviews. BMJ Open.

[20] Lewis CC, Klasnja P, Powell BJ, Lyon AR, Tuzzio L, Jones S (2018). From classification to causality: advancing understanding of mechanisms of change in implementation science. Front Public Health.

[21] Lewis CC, Boyd MR, Walsh-Bailey C, Lyon AR, Beidas R, Mittman B (2020). A systematic review of empirical studies examining mechanisms of implementation in health. Implement Sci.

[22] Sarkies MN, Francis-Auton E, Long JC, Pomare C, Hardwick R, Braithwaite J (2022). Making implementation science more real. BMC Med Res Methodol.

[23] Denis J-L, Hébert Y, Langley A, Lozeau D, Trottier L-HJ (2002). Explaining diffusion patterns for complex health care innovations. Health Care Manag Rev.

[24] Hawe P, Shiell A, Riley T (2004). Complex interventions: how “out of control” can a randomised controlled trial be?. BMJ.

[25] Lennox L, Barber S, Stillman N, Spitters S, Ward E, Marvin V (2022). Conceptualising interventions to enhance spread in complex systems: a multisite comprehensive medication review case study. BMJ Qual Saf.

[26] Kirk MA, Haines ER, Rokoske FS, Powell BJ, Weinberger M, Hanson LC (2021). A case study of a theory-based method for identifying and reporting core functions and forms of evidence-based interventions. Transl Behav Med.

[27] Horton TJ, Illingworth JH, Warburton WH (2018). Overcoming challenges in codifying and replicating complex health care interventions. Health Aff.

[28] Whitaker RG, Sperber N, Baumgartner M, Thiem A, Cragun D, Damschroder L (2020). Coincidence analysis: a new method for causal inference in implementation science. Implement Sci.

[29] Ragin CC. Using qualitative comparative analysis to study causal complexity. Health Serv Res. 1999;34:1225–39. PMC1089061 10591281

[30] The AHSN Network. The AHSN Network. (2019). https://www.ahsnnetwork.com. Accessed 18 Mar 2022.

[31] Rihoux B, De Meur G. Crisp-set qualitative comparative analysis (csQCA). In Configurational compartive methods. Qualitative comparative analysis (QCA) and related techniques. Edited by Rihoux B, Ragin C. Thousand Oaks: Sage; 2009:33–68.

[32] Schneider CQ, Wagemann C. Standards of good practice in Qualitative Comparative Analysis (QCA) and fuzzy-sets. Comp Sociol. 2010;9(3):397–418.

[33] Sibley A, Ziemann A, Robens S, Scarbrough H, Tuvey S: Review of spread and adoption approaches across the AHSN network. The AHSN Network; 2021.

[34] Ferlie E, Nicolini D, Ledger J, D’Andreta D, Kravcenko D, de Pury J (2017). NHS top managers, knowledge exchange and leadership: the early development of academic health science networks – a mixed-methods study.

[35] The AHSN Network: Transfers of Care Around Medicines (TCAM). https://www.ahsnnetwork.com/about-academic-health-science-networks/national-programmes-priorities/transfers-care-around-medicines-tcam (2019). Accessed 18 Mar 2022.

[36] Nazar H, Brice S, Akhter N, Kasim A, Gunning A, Slight SP (2016). New transfer of care initiative of electronic referral from hospital to community pharmacy in England: a formative service evaluation. BMJ Open.

[37] QSR International Pty Ltd: NVivo. https://www.qsrinternational.com/nvivo-qualitative-data-analysis-software/home (2020). Accessed 18 Mar 2022.

[38] Damschroder LJ, Aron DC, Keith RE, Kirsh SR, Alexander JA, Lowery JC (2009). Fostering implementation of health services research findings into practice: a consolidated framework for advancing implementation science. Implement Sci.

[39] Sarkies M, Long JC, Pomare C, Wu W, Clay-Williams R, Nguyen HM (2020). Avoiding unnecessary hospitalisation for patients with chronic conditions: a systematic review of implementation determinants for hospital avoidance programmes. Implement Sci.

[40] Northridge ME, Metcalf SS (2016). Enhancing implementation science by applying best principles of systems science. Health Res Policy Syst.

[41] Yakovchenko V, Miech EJ, Chinman MJ, Chartier M, Gonzalez R, Kirchner JE (2020). Strategy configurations directly linked to higher hepatitis C virus treatment starts: an applied use of configurational comparative methods. Med Care.

[42] TCAM Spread data. National Metrics Dashboard. The AHSN Network. 2020. Accessed 26 Oct 2020.

[43] Cronquist L: Tosmana (v 1.61). Trier University of Trier. https://www.tosmana.net. (2019). Accessed 18 Mar 2022.

[44] MacKillop E, Quarmby S, Downe J (2020). Does knowledge brokering facilitate evidence-based policy? A review of existing knowledge and an agenda for future research. Policy Polit.

[45] Harvey G, Llewellyn S, Maniatopoulos G, Boyd A, Procter R (2018). Facilitating the implementation of clinical technology in healthcare: what role does a national agency play?. BMC Health Serv Res.

[46] Ferlie E, Fitzgerald L, Wood M, Hawkins C (2005). The nonspread of innovations: the mediating role of professionals. Acad Manag J.

[47] Proctor EK, Powell BJ, McMillen JC (2013). Implementation strategies: recommendations for specifying and reporting. Implement Sci.

[48] Ilott I, Gerrish K, Eltringham SA, Taylor C, Pownall S (2016). Exploring factors that influence the spread and sustainability of a dysphagia innovation: an instrumental case study. BMC Health Serv Res.

[49] Antons D, Piller FT (2015). Opening the black box of “not invented here”: attitudes, decision biases, and behavioral consequences. Acad Manag Perspect.

[50] Gabbay J, Le May A, Pope C, Brangan E, Cameron A, Klein JH (2020). Uncovering the processes of knowledge transformation: the example of local evidence-informed policy-making in United Kingdom healthcare. Health Res Policy Syst.

[51] Turner S, D´Lima D, Sheringham J, Swart N, Hudson E, Morris S, Fulop NJ (2022). Evidence use as sociomaterial practice? A qualitative study of decision-making on introducing service innovations in health care. Public Manag Rev.

[52] Rhodes T, Lancaster K (2019). Medicine: evidence-making interventions in health: a conceptual framing. Soc Sci Med.

[53] Kane H, Lewis MA, Williams PA, Kahwati LC. Using Qualitative Comparative Analysis to understand and quantify translation and implementation. Transl Behav Med. 2014;4:201–8. 10.1007/s13142-014-0251-6 PMC4041929 24904704

