# Supplementary material for: Identifying core strategies and mechanisms for spreading a national medicines optimisation programme across England—a mixed-method study applying qualitative thematic analysis and Qualitative Comparative Analysis
Source: Implement Sci Commun. 2022 Oct 29;3:116. doi: 10.1186/s43058-022-00364-5 (PMC9617223; doi:10.1186/s43058-022-00364-5)
Supplement: Supplementary file 2 — Additional file 2. Interview guide. [file 43058_2022_364_MOESM2_ESM.pdf]

Identifying core strategies and mechanisms for spreading a national medicines optimisation programme across England - A mixed-method study applying qualitative thematic analysis and Qualitative Comparative Analysis

Additional file 2

Interview guide

1. What is your role in, and experience of, spread and adoption work at your AHSN?

2. Reflecting on your experience of working across different projects in the last two years, does your AHSN have a shared approach to encouraging the spread and adoption of innovations? If not, how do approaches vary?

3. How did your AHSN / you develop your spread and adoption approaches (evidence/theory use, adaptation to existing approaches)?

4. Could you talk us through how your spread and adoption work looks like in practice (for the national spread programme)?

5. What does a successful and an unsuccessful case of spread and adoption work look like for you (for the national spread programme)?

6. In your experience what are the key barriers and enablers to your spread and adoption work (for the national spread programme)?

7. In your experience, how have national policy frameworks/documents influenced your spread and adoption work (for the national spread programme)?

8. What are the main lessons you took from your spread and adoption work (for the national spread programme)? Is there anything you would change about the spread and adoption approaches at your AHSN for the future?

9. Do you have any other views about spread and adoption approaches you would like to share with us?
