# Supplementary material for: Identifying core strategies and mechanisms for spreading a national medicines optimisation programme across England—a mixed-method study applying qualitative thematic analysis and Qualitative Comparative Analysis
Source: Implement Sci Commun. 2022 Oct 29;3:116. doi: 10.1186/s43058-022-00364-5 (PMC9617223; doi:10.1186/s43058-022-00364-5)
Supplement: Supplementary file 3 — Additional file 3. Coding tree. [file 43058_2022_364_MOESM3_ESM.pdf]

Identifying core strategies and mechanisms for spreading a national medicines optimisation programme across England - A mixed-method study applying qualitative thematic analysis and Qualitative Comparative Analysis

Additional file 3

Coding tree

Spread strategies and mechanisms

Category<br><br>(derived from Powell et al. [15] and Leeman et al. [12])	Codes
High-level adoption and spread strategies	Honest (realistic) broker
	AHSN facilitator and supporter role
	Not a sales force – local needs view over innovation view
	Equality
	Function over form
	Pathway change vs technolgy focus
	Flexibility in applying spread approach
	Adaptation of the innovation to the local context
	Varied staff skills set and background
	Scale-out
	Achieving sustainability
Planning/preparatory strategies	Staff expertise / match (AHSN) staff to task by expertise
	Relationship building, stakeholder engagement
	(Senior) clinical expertise and support
	System-level steering group/decision-making / implementing at system level
	Engaging all relevant stakeholders from the start
	Champions
	Ownership
	Tapping into, building on existing network, building new networks
	Based on existing/previous work/relationships in area
	Intersectoral collaboration

	Baselining, needs assessment, system understanding
	Understanding originating context
Financing strategies	Seed funding, pump-priming, back-fill
	Cash vs human resources
	Funding small operational things in health system
	Business plan development
	Funding local demonstrator
	External / additional funding
Structural strategies	AHSN resources
	Pharmacist as staff
Quality/project management strategies	Quality/project management/methods/skills
	Step-by-step vs metrics-focused
	Personalised support
	Regular check-in, team meetings
	Providing evidence
	Demonstrator cases, pilots
	Evaluation/monitoring
	Qualitative & quantitative metrics
	‘Work with the willing’
	Certificate of attendance
	Escalation
	Enthusiasm
	Patient and Public Involvement (PPI)
	Memorandum of Understanding (MoU)
Dissemination/communication strategies	Communication methods
	Tailoring language
	Storytelling
	Patient information
	Communicate and celebrate successes
	Hearts and minds
	Launch event

Capacity building/education strategies	Implementation packs, toolkits
	Case studies
	Local system peer network, sharing, learning
	Community of practice
	Online training
	Training for stakeholders
	Events
	Peer support, learning from other AHSN
	Continuous learning

Contextual determinants

**Category** **(derived from Damschroder et al. [33])**	**Codes**
Individual stakeholder characteristics	Staff turnover
	Time/availability
	Innovation mindset
	Non-health stakeholders
Innovation characteristics	Technology/software characteristics
	Evidence base
	Cost-efficiency
	Perverse incentives/unintended consequences
	Timescales (time until benefits are realised)
	Innovator/supplier characteristics
Outer context characteristics (regional)	Information governance
	Antecedents/alternatives in place
	Health system organisation changes, provider organisation changes
	Complexity, variation health system organisation
	Benefits realised in other part of the (health) system
	Data availability
	Competing demands/priorities

	Silo-thinking
	Media
Outer context characteristics (national)	National policies (e.g., NHS Long Term Plan)
	National professional membership organisation (e.g., Royal Colleges)
	National government agency (e.g., Public Health England)
	National charities
	National data sets
	National, mandatory contractual arrangements
	Alignment with/utilisation for national quality assurance programmes and regulations (e.g., Quality Outcomes Framework)
	Selection process of national AHSN spread programmes
	National vs local priorities
	AHSN national spread programme metrics
	Time plans of AHSN national spread programme
	Flexibility of AHSN national spread programme
	Support, resources provided by AHSN national spread programme
	Innovation Portal
