# Supplementary material for: Identifying core strategies and mechanisms for spreading a national medicines optimisation programme across England—a mixed-method study applying qualitative thematic analysis and Qualitative Comparative Analysis
Source: Implement Sci Commun. 2022 Oct 29;3:116. doi: 10.1186/s43058-022-00364-5 (PMC9617223; doi:10.1186/s43058-022-00364-5)
Supplement: Supplementary file 4 — Additional file 4. TCAM spread outcomes. [file 43058_2022_364_MOESM4_ESM.pdf]

Identifying core strategies and mechanisms for spreading a national medicines optimisation programme across England - A mixed-method study applying qualitative thematic analysis and Qualitative Comparative Analysis

Additional file 4

TCAM spread outcomes (derived from AHSN Network National Metrics Dashboard [41])

AHSN	Adoption rate [%]			Change Q3 18/19 - Q3 19/20 [%]	Change Q3 19/20 - Q3 20/21 [%]
	Q3 18/19	Q3 19/20	Q2 20/21		
Oxford	0	20	20	20	0
Health Innovation Manchester	9	33	44	24	11
West Midlands	0	20	60	20	40
East Midlands	50	50	63	0	13
HIN	14	14	29	0	15
Yorkshire & Humber	21	57	57	36	0
KSS	10	30	50	20	20
South West	43	57	57	14	0
Innovation Agency	47	56	56	9	0
NENC	44	25	25	-19	0
Eastern	18	45	60	27	15
UCLP	7	33	29	26	-4
Wessex	50	63	63	13	0
West of England	83	50	100	-33	50
ICHP	17	50	67	33	17
Total	25	40	51	15	11

Legend

no change
increase
increase > 25% points
decrease

unsuccessful <50% adoption
successful >= 50% adoption

Q3 18/19 = Oct-Dec 2018
Q3 19/20 = Oct-Dec 2019
Q2 20/21 = Jul-Sep 2020
(latest available data 26/10/20)
