# Supplementary material for: Identifying core strategies and mechanisms for spreading a national medicines optimisation programme across England—a mixed-method study applying qualitative thematic analysis and Qualitative Comparative Analysis
Source: Implement Sci Commun. 2022 Oct 29;3:116. doi: 10.1186/s43058-022-00364-5 (PMC9617223; doi:10.1186/s43058-022-00364-5)
Supplement: Supplementary file 5 — Additional file 5. Research team and reflexivity. [file 43058_2022_364_MOESM5_ESM.pdf]

Identifying core strategies and mechanisms for spreading a national medicines optimisation programme across England - A mixed-method study applying qualitative thematic analysis and Qualitative Comparative Analysis

Additional file 5

Research team and reflexivity

The research team consisted of two researchers based at City, University of London (AZ, PhD, female, senior research fellow, and HS, PhD, male, full professor) and three researchers based at two AHSNs, Wessex AHSN (AS, PhD, male, evaluation programme manager) and South West AHSN (ST, MSc/PhD, male, evaluation researcher, and SR, PhD, female, evaluation lead and spread fellow). AHSN research team members added a unique inside-perspective and understanding concerning general spread activity in an AHSN environment that was complemented with the external view brought in by the university-based research team members. Research team members had no prior relationship to participants they recruited or interviewed. Research team members briefly introduced themselves (i.e., name, affiliation, occupation, role in study) and the goals of the study to participants during recruitment and again at the beginning of the interviews. To avoid pressure on participants and reduce risk of bias, AS and ST did not recruit participants, conduct interviews or extract data related to their own AHSNs. No research team member was involved in TCAM spread work. The research team have a health services and social science background with AS and AZ applying an implementation science lens and HS applying an organisation studies lens to innovation research. All research team members have training and experience in applying qualitative methods and conducting semi-structured interviews. AZ and HS have experience applying QCA.
