# Supplementary material for: Identifying core strategies and mechanisms for spreading a national medicines optimisation programme across England—a mixed-method study applying qualitative thematic analysis and Qualitative Comparative Analysis
Source: Implement Sci Commun. 2022 Oct 29;3:116. doi: 10.1186/s43058-022-00364-5 (PMC9617223; doi:10.1186/s43058-022-00364-5)
Supplement: Supplementary file 6 — Additional file 6. Case descriptions. [file 43058_2022_364_MOESM6_ESM.pdf]

Identifying core strategies and mechanisms for spreading a national medicines optimisation programme across England - A mixed-method study applying qualitative thematic analysis and Qualitative Comparative Analysis

Additional file 6

Summary case descriptions

AHSN ID	TCAM spread success (Additional file 4)	Key contextual determinants	Key/focus spread strategies/mechanisms	Key lessons
**01**	No	• Trust's IT restructure<br>• Limited capacity stakeholders<br>• Lack of financial security long-term<br>• Late contractual arrangements<br>• Limited national level and supplier support due to delayed start	• Delayed start (late appointment AHSN staff)<br>• Negative past experiences adopting TCAM<br>• Application of AHSN's high-level framework (adjusted to start at later stage in framework)<br>• Setup of senior project group setup and steering groups for each site<br>• Intersectoral engagement (pharmacists, senior trust management, trust's IT and IG departments, commissioners, local pharmaceutical committees)<br>• Newsletters for community pharmacists	• More senior-level support (e.g., to engage IT departments)<br>• Provision of local evidence on benefits for trusts and limited extra staff workload using TCAM important

**02**	No	• Trust IT department capacity	• Delayed start (capacity AHSN)<br>• Application of AHSN's high-level framework (adjusted to start at later stage in framework)<br>• Employment of local chief pharmacist at AHSN<br>• Senior whole-system governance process<br>• Use of local networks (chief trust pharmacists and CCG pharmacists)<br>• Scale-out to include more conditions<br>• Developed training/guidance videos for stakeholders	• Earlier employment of pharmacist at AHSN<br>• Whole system senior-level support very helpful<br>• Obtaining additional local financial support helpful
**03**	No	• Trusts' IT readiness	• Delayed start (lack of pharmacist expertise at AHSN)<br>• Previous local adoption of TCAM increased readiness for adoption during national programme<br>• Long time to process IG sign-off at trusts<br>• Employment of local chief pharmacist at AHSN<br>• Shared learning with other AHSNs and adaptation of other AHSNs' materials<br>• Implement at system level (STP)<br>• Use of local networks (system-wide pharmacy and medicines optimisation group, trust committees)	• Earlier employment of pharmacist at AHSN<br>• IG sign-off might have been easier with national endorsement (NHS Digital) and national IG toolkit<br>• Project management training for clinicians at AHSNs would have been helpful

			• Involvement of GPs (via Local Medical Committee)<br>• Adaptation to implement in community and mental health trusts instead of acute trusts and focus on different patient cohorts<br>• Launch first with LPC, then trusts	
**04**	Yes	• Capacity, IT readiness trusts	• Employment of local deputy chief pharmacist at AHSN<br>• Use of local networks (STP groups)<br>• Engage chief operating officers in trusts to increase IT Department engagement<br>• Organising events in evening hours around locally relevant topics, not only TCAM<br>• Pump-primed trusts to pay for implementation and licenses instead of paying for licenses directly	• Focus more on planning for sustainability, not just initial adoption (also at national level)<br>• Shared learning across local system helpful (especially with local adopter/ demonstrator)

**05**	No	• Trust’s capacity	• AHSN key methodology of ‘community of practice’ not applied to TCAM spread<br>• Focus on behavioural change activities and keeping up motivation and enthusiasm of stakeholders<br>• Focus on establishing multi-stakeholder/intersectoral discussions<br>• Celebrating successes with stakeholders (milestones)<br>• Focus on storytelling approaches	• Spread skills and knowledge at AHSN helpful<br>• More collaboration with CCGs<br>• Stepped and locally adjusted national targets (lower targets at beginning increase motivation, take into account different local priorities and readiness)<br>• Add process metrics (e.g., relationship building)<br>• Increase national programme time (> 2 years, initial relationship building takes a long time)

**6**	Yes	• Local stakeholders ‘liked’ TCAM<br>• Capacity, IG process trusts	• Previous local adoption of TCAM<br>• System-level/wide implementation (ICS)<br>• Co-creation with local stakeholders (let ICS set target, trust/LPC set patient cohort and develop local forms)<br>• Setup of TCAM intersectoral community of practice (each trust, LPC, PCN, CCG)<br>• Developed IG FAQ<br>• Use local networks<br>• Include patient’s voice, focus on storytelling<br>• Stepped approach (formulated lower local targets at beginning, increased later)<br>• Developed online training<br>• Celebrate and communicate successes (newsletter, poster development)<br>• Development sustainability plan<br>• Development/write-up of case studies on different patient cohorts<br>• Development of evaluation toolkit	• Do not use benchmarking (blame and shame) but work with laggards and create enthusiasm based on variation in output<br>• Involving all stakeholders from start proved useful

**07**	No	• Capacity and mindset stakeholders<br>• Local stakeholders ‘liked’ TCAM<br>• Lack of financial security long-term<br>• Late contractual arrangements	• Limited resources at AHSN (generally)<br>• System-level approach (STP)<br>• intersectoral stakeholder involvement and shared partnership working<br>• Use of local networks (medicine optimisation programme boards) and invite other local stakeholders (e.g., trusts) to join board meetings<br>• Training and events for community pharmacy<br>• Build on existing trusted relationships<br>• Keeping on agenda (regular check-in)<br>• Project teams and TCAM leads in each trust, and inviting community pharmacy to join team<br>• Regular meetings with CCGs, GPs<br>• Created dashboard with referral information and regular sharing with all local stakeholders	• More local leadership/ champions<br>• Learning from other AHSNs (early adopters) helpful<br>• Engage with community pharmacy early<br>• Initial engagement and relationship-building takes time

**08**	Yes	• IT restructure trust<br>• Trust champion left	• Previous local adoption of TCAM<br>• Employed part-time local pharmacist by AHSN<br>• Engaged with CCG Medicines Optimisation teams<br>• Use of local networks (e.g., local pharmacy networks)<br>• Published evaluation of early adopter case<br>• Use patient voice/analogies, storytelling<br>• Communicate successes	• More intersectoral/system-wide engagement and shared learning activities (e.g., action learning set/ community of practice)<br>• Increase national programme time (> 2<br>• years to ensure supporting sustainability)<br>• Scale within stakeholder organisation (involve more than 1-2 champions/<br>• responsible people, involve teams)<br>• Apply process metrics (include process milestones)
**09**	Yes	• Capacity, IT readiness, IG processes trusts<br>• Staff turnover<br>• Stakeholders 'liked' TCAM	• Previous local adoption of TCAM<br>• Employed local pharmacist by AHSN<br>• QI/coaching/support methodologies<br>• Use of networks<br>• Use of patient voice<br>• Senior-level support (engage regional IT CIOs to engage IT department)	• More clinical champions in each trust<br>• More senior-level support<br>• Patient safety/patient voice important driver<br>• Start with system/ stakeholder map

**10**	No	• Misconception barriers and teething problems that have been overcome by now<br>• Lack of trust in evidence	• Previous local adoption of TCAM (created competitiveness/lack of ownership in local system)<br>• Project manager at AHSN with experience working in pharma-context (but no pharmacist)	• More senior-level support<br>• More champions in each trust
**11**	No	• Trust IT readiness, capacity, accountability<br>• Worries of community pharmacists	• Trust-by-trust engagement and network engagement with LPCs<br>• Intersectoral meetings (LPC, trust, supplier),<br>• Co-production (definition of patient cohort and condition focus)<br>• Community pharmacy engagement event in evening (AHSN pays event costs)<br>• Training through pharmacists at hospital (train the trainer through AHSN)<br>• Development of material, training videos<br>• Poster creation for wards with option to update day-by-day progress<br>• Regular lessons learned workshops<br>• Support for stakeholders to bid for additional funding	• Stepped approach (start with small cohort/ department, then scale later)<br>• Setup of steering group inviting every LPC, trust, supplier, patient<br>• representatives, and CCG planned

**12**	No	• Capacity stakeholders, especially IT Department trust<br>• Financial commitment trusts<br>• Financial uncertainty community pharmacy	• Delayed start (stakeholders questioning evidence, negative past experiences with other adoption projects)<br>• Employed pharmacist at AHSN<br>• Generated literature review to add to evidence base	• More financial incentives to run demonstrators<br>• Initial engagement takes time
**13**	Yes	• Software known to/ used by stakeholders<br>• Capacity stakeholders	• Employed pharmacist at AHSN<br>• Intersectoral, senior advisory group (pharmacists, GPs, patients)<br>• Use of networks<br>• Patient voice focus	• PPI/patient voice impactful<br>• Engagement with CCGs important<br>• Baselining/initial engagement takes time
**14**	Yes	• Evidence challenged<br>• Staff turnover<br>• Trust IT restructure	• Previous local adoption of TCAM<br>• Intersectoral steering group (trusts, CCGs, LPCs, other community services)<br>• Patient voice/narrative<br>• Test runs referring patients to community pharmacy	• Scale-out to mental health trusts, care homes and other patient cohorts planned<br>• Incorporating patient voice impactful<br>• Continuous evaluation essential<br>• Involving all stakeholders from the beginning crucial

**15**	Yes	• Stakeholders had no prior knowledge of software<br>• Referrals limited to own AHSN service area	• Employed pharmacist at AHSN<br>• Use local network (medicine optimisation/pharmacy network)<br>• MoU with stakeholders (sustainability)<br>• Community of practice approach<br>• Patient voice/narrative<br>• Online training<br>• (Co-)production of communication packs<br>• Setup of pan-regional AHSNs group for shared learning and cooperation	• More sharing of local evaluation data with stakeholders<br>• Less hands-on facilitation<br>• More patient and public involvement<br>• More involvement CCGs<br>• More use of stakeholders with research background in local evaluation<br>• Initial engagement takes time

AHSN = Academic Health Science Network, CCG = Clinical Commissioning Groups, CIO = Chief Information Officer, FAQ = Frequently Asked Questions, GP = General practitioner, ICS = Integrated Care System, ID = Identifier, IG = Information governance, IT = Information technology, LPC = Local Pharmaceutical Committee, NHS = National Health System, PCN = Primary Care Network, STP = Sustainability and Transformation Plans, TCAM = Transfer of Care Around Medicines
