# Supplementary material for: Identifying core strategies and mechanisms for spreading a national medicines optimisation programme across England—a mixed-method study applying qualitative thematic analysis and Qualitative Comparative Analysis
Source: Implement Sci Commun. 2022 Oct 29;3:116. doi: 10.1186/s43058-022-00364-5 (PMC9617223; doi:10.1186/s43058-022-00364-5)
Supplement: Supplementary file 7 — Additional file 7. Detailed QCA results. [file 43058_2022_364_MOESM7_ESM.pdf]

**Identifying core strategies and mechanisms for spreading a national medicines optimisation programme across England - A mixed-method study applying qualitative thematic analysis and Qualitative Comparative Analysis**

**Additional file 7**

**Detailed results of the Qualitative Comparative Analysis (QCA)**

Created with Tosmana Version 1.6.1

**Successful spread**

Necessity of conditions

| Condition | Necessary Sufficiency |
|---|---|
| **TIMELY** | **1,0000** |
| **timely** | **0,0000** |
| ADOPT | 0,7143 |
| adopt | 0,2857 |
| **PHARMA** | **1,0000** |
| **pharma** | **0,0000** |
| INTERSECT | 0,5714 |
| intersect | 0,4286 |

Minimisation (parsimonious and conservative/complex solution)

*Prime implicants*

| Prime implicants | Consistency |
|---|---|
| **TIMELY * PHARMA** | **1.0** |
| **TIMELY** * adopt * intersect | 1.0 |
| **TIMELY** * ADOPT * INTERSECT | 1.0 |
| ADOPT * **PHARMA** * intersect | 1.0 |

*Simplifying assumptions (0)*

*Solution*

**TIMELY*PHARMA** (Consistency 1.0, Coverage 1.0, Unique Coverage 1.0) (cases: 4 + 6,13,14 + 8,9 +15)

*Venn diagram*

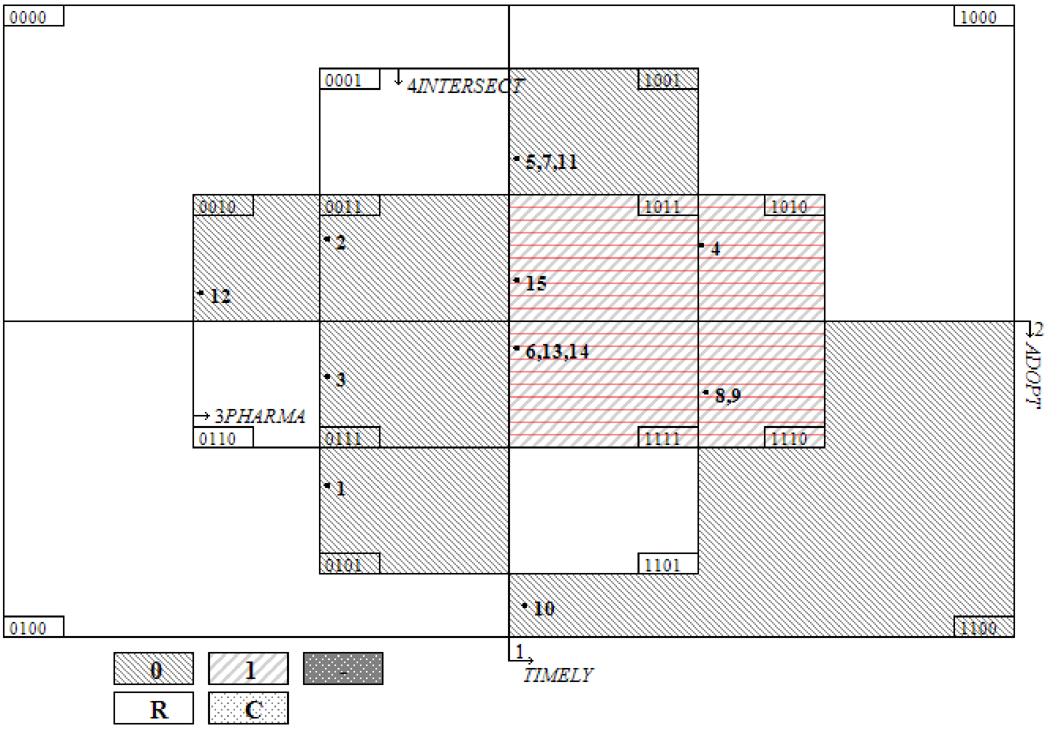


Red, horizontal lines: solution (TIMELY*PHARMA)

*Case example*

AHSN 04

| AHSN ID | **TIMELY** | ADOPT | **PHARMA** | INTERSECT | OUTCOME | Raw Consistency |
|---|---|---|---|---|---|---|
| 4 | **1** | 0 | **1** | 0 | 1 | 1 |

This AHSN employed a local deputy chief pharmacist to lead the spread of TCAM in their area. There was no delay in starting the spread work. This case shows how the two strategies/mechanisms (QCA codes: TIMELY, PHARMA) are more relevant to lead to success than having adopted TCAM beforehand (QCA code ADOPT) and applying an intersectoral approach (QCA code INTERSECT), both of which this AHSN didn't engage in (condition value = 0).

**Unsuccessful spread**

*Necessity of conditions*

| Unsuccessful spread (outcome = 0) | |
|---|---|
| Condition | Necessary Sufficiency |
| **TIMELY** | **0,5000** |
| **timely** | **0,5000** |
| ADOPT | 0,3750 |
| adopt | 0,6250 |
| **PHARMA** | **0,3750** |
| **pharma** | **0,6250** |
| INTERSECT | 0,7500 |
| intersect | 0,2500 |

Minimisation (conservative/complex solution)

*Prime implicants*

| Prime implicants | Consistency |
|---|---|
| **timely** * ADOPT * INTERSECT | 1.0 |
| **timely** * PHARMA * INTERSECT | 1.0 |
| **timely** * adopt * PHARMA | 1.0 |
| TIMELY * adopt * **pharma** * INTERSECT | 1.0 |
| TIMELY * ADOPT * **pharma** * intersect | 1.0 |

*Solutions*

**timely** * ADOPT * INTERSECT +
**timely** * adopt * PHARMA +
TIMELY * adopt * **pharma** * INTERSECT +
TIMELY * ADOPT * **pharma** * intersect
(Consistency: 1.0, Coverage: 1.0)

**timely** * ADOPT * INTERSECT (Consistency: 1.0, Coverage: 0.25, Unique Coverage: -)
(cases: 1+3)

**timely** * adopt * PHARMA (Consistency: 1.0, Coverage: 0.25, Unique Coverage: -)
(cases: 2+12)

TIMELY * adopt * **pharma** * INTERSECT (Consistency: 1.0, Coverage: 0,375, Unique Coverage: -)
(cases: 5,7,11)

TIMELY * ADOPT * **pharma** * intersect (Consistency: 1.0, Coverage: 0.125, Unique Coverage: -)
(case: 10)

*Venn diagram*

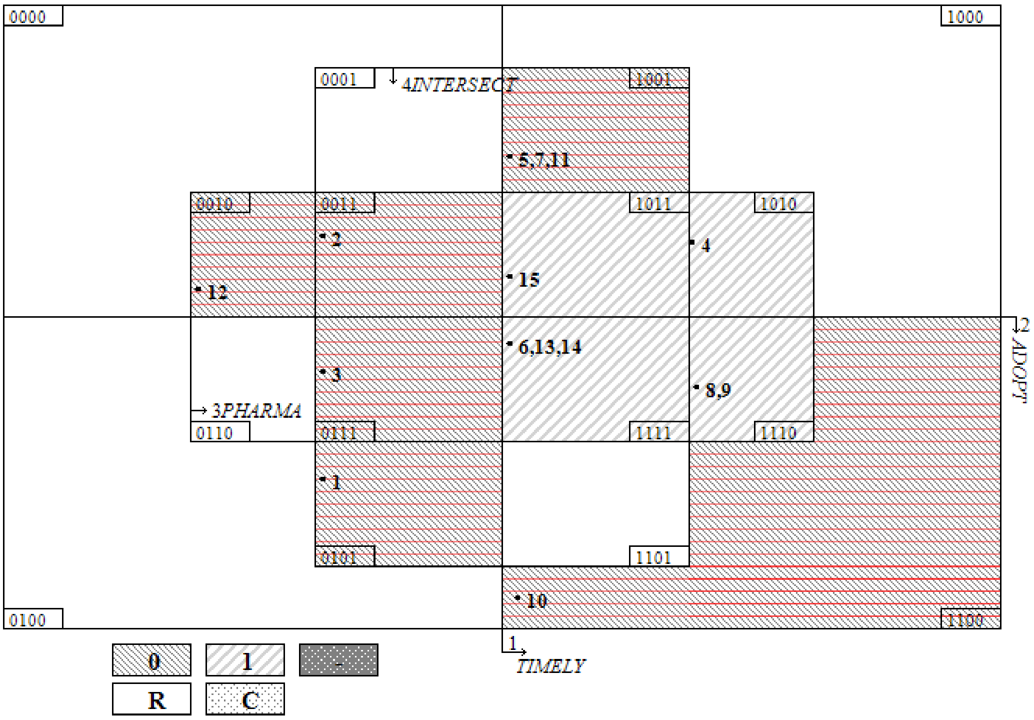


Red, horizontal lines: solution (timely * ADOPT * INTERSECT + timely * adopt * PHARMA + TIMELY * adopt * pharma * INTERSECT + TIMELY * ADOPT * pharma * intersect)

## Minimisation (parsimonious solution)

*Prime implicants*

| Prime implicants | Consistency |
|---|---|
| **timely** | **1.0** |
| **pharma** | **1.0** |

*Simplifying assumptions (6)*

TIMELY{0}ADOPT{0}PHARMA{0}INTERSECT{0} +
TIMELY{0}ADOPT{0}PHARMA{0}INTERSECT{1} +
TIMELY{0}ADOPT{1}PHARMA{0}INTERSECT{0} +
TIMELY{0}ADOPT{1}PHARMA{1}INTERSECT{0} +
TIMELY{1}ADOPT{0}PHARMA{0}INTERSECT{0} +
TIMELY{1}ADOPT{1}PHARMA{0}INTERSECT{1}

*Solutions*

**timely + pharma** (Consistency: 1.0, Coverage: 1.0)

**timely** (Consistency: 1.0, Coverage: 0.5, Unique Coverage: 0.375)

(cases: 1+2+3+12)

**pharma** (Consistency: 1.0, Coverage: 0.625, Unique Coverage: 0.5)

(cases: 1+5,7,11+10)

*Venn diagram*

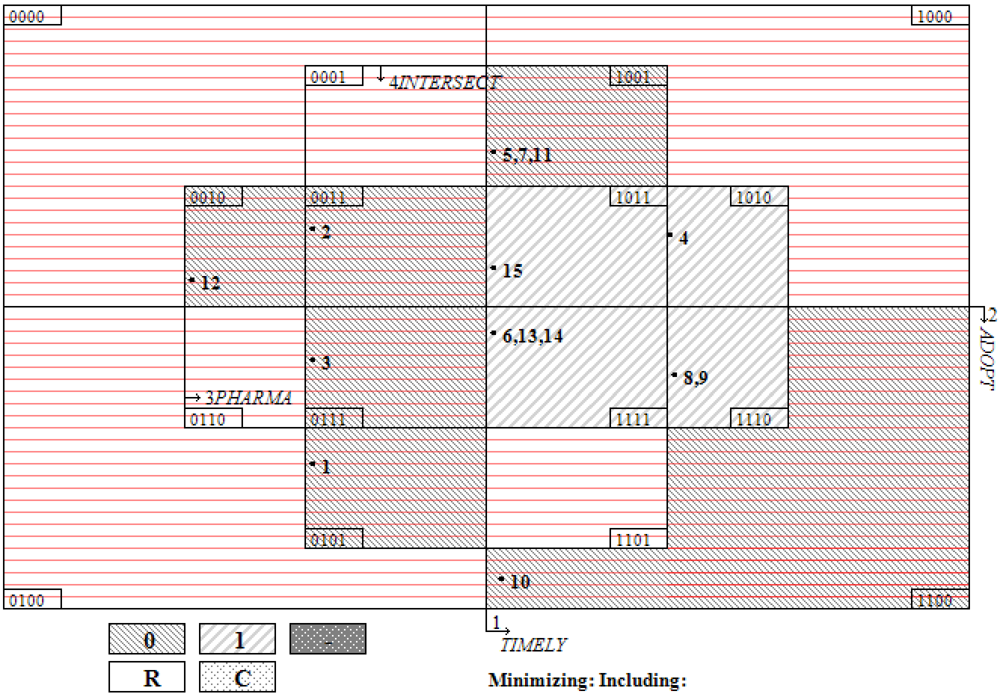


Red, horizontal lines: solution (timely + pharma)

*Case example*

AHSN 03

| AHSN ID | **TIMELY** | ADOPT | **PHARMA** | INTERSECT | OUTCOME | Raw Consistency |
|---|---|---|---|---|---|---|
| 3 | **0** | 1 | **1** | 1 | 0 | 0 |

The AHSN lacked pharmaceutical expertise in their team which they described as one key reason for unsuccessful spread of TCAM in their area. They employed a pharmacist (QCA code: PHARMA) but only late in the course of the national programme which led to a delayed start (QCA code: timely). The case shows that the absence of the combination of these two spread strategies/mechanisms led to an unsuccessful spread. It also shows that the combination of these two strategies/mechanisms are more important than having adopted TCAM before the start of the national programme nor an intersectoral engagement approach which were both applied in this case.
