# Supplementary material for: Identifying core strategies and mechanisms for spreading a national medicines optimisation programme across England—a mixed-method study applying qualitative thematic analysis and Qualitative Comparative Analysis
Source: Implement Sci Commun. 2022 Oct 29;3:116. doi: 10.1186/s43058-022-00364-5 (PMC9617223; doi:10.1186/s43058-022-00364-5)
Supplement: Supplementary file 8 — Additional file 8. Policy and practice implications. [file 43058_2022_364_MOESM8_ESM.pdf]

Identifying core strategies and mechanisms for spreading a national medicines optimisation programme across England - A mixed-method study applying qualitative thematic analysis and Qualitative Comparative Analysis

Additional file 8

Policy and practice implications

The identification of core strategies/mechanisms and pre-conditional and mediating contextual determinants for a particular innovation offers those facilitating spread and adopting and implementing innovations a priority list for tailoring activities to ensure successful spread. For TCAM and similar innovations, the employment of a local, senior pharmacist as spread facilitator combined with a timely start make up the core spread strategy which should be prioritised over other activities. Further, strategies can be prioritised and tailored to engage pre-conditional and mediating contextual determinants that are linked to enable successful spread, in this case contractual arrangements, funding, and localised evidence. The core mediating factor of contractual arrangements identified for TCAM is only partially in the control of the AHSN. There is a role for policymakers at the national level to understand the importance of some of the contextual factors which are in their control to contribute to better aligning contractual arrangements to enable the spread of a national innovation. We recommend that spread facilitators such as AHSNs apply comparative case study designs as part of formative process evaluations early in the implementation and spread process to identify core strategies/mechanisms and preconditional and mediating contextual determinants and their causal links to successful spread to inform the future wider spread process across diverse contexts.
